# Comparative evaluation of three interfaces for non-invasive ventilation: a randomized cross-over design physiologic study on healthy volunteers

**DOI:** 10.1186/cc13175

**Published:** 2014-01-03

**Authors:** Rosanna Vaschetto, Audrey De Jong, Matthieu Conseil, Fabrice Galia, Martin Mahul, Yannael Coisel, Albert Prades, Paolo Navalesi, Samir Jaber

**Affiliations:** 1 Anesthesia and Intensive Care Medicine, Maggiore della Carità Hospital, Novara, Italy; 2 Intensive Care Unit and Transplantation, Critical Care and Anesthesia Department (DAR B), Hôpital Saint-Éloi, CHU de Montpellier, Montpellier, France; 3 Dipartimento di Medicina Traslazionale, Università del Piemonte Orientale “Amedeo Avogadro”, Alessandria-Novara-Vercelli, Italy; 4 Anesthesia and Intensive Care Medicine, Sant’Andrea Hospital, Vercelli, Italy; 5 CRRF Mons. L. Novarese, Moncrivello, Vercelli, Italy; 6 Department of Anesthesia and Critical Care B (DAR B), Hôpital Saint Eloi, 80, avenue Augustin Fliche, 34295 Montpellier, Cedex 5, France

## Abstract

**Introduction:**

Interface choice is crucial for non-invasive ventilation (NIV) success. We compared a new interface, the helmet next (H_N_), with the facial mask (FM) and the standard helmet (H_S_) in twelve healthy volunteers.

**Methods:**

In this study, five NIV trials were randomly applied, preceded and followed by a trial of unassisted spontaneous breathing (SB). Baseline settings, for example, 5 cmH_2_O of both inspiratory pressure support (PS) and positive end-expiratory pressure (PEEP), were applied through FM, H_S_ and H_N_, while increased settings (PS and PEEP of 8 cmH_2_O) were only applied through H_S_ and H_N_. We measured flow, airway, esophageal and gastric pressures, and calculated inspiratory effort indexes and trigger delays. Comfort was assessed with a visual-analog-scale.

**Results:**

We found that FM, H_S_ and H_N_ at baseline settings were not significantly different with respect to inspiratory effort indexes and comfort. Inspiratory trigger delay and time of synchrony (TI,synchrony) were significantly improved by FM compared to both helmets, whereas expiratory trigger delay was shorter with FM, as opposed to H_S_ only. H_N_ at increased settings performed better than FM in decreasing inspiratory effort measured by pressure-time product of transdiaphragmatic pressure (PTPdi)/breath (10.7 ± 9.9 versus 17.0 ± 11.0 cmH_2_O*s), and PTPdi/min (128 ± 96 versus 204 ± 81 cmH_2_O*s/min), and PTPdi/L (12.6 ± 9.9 versus 30.2 ± 16.8 cmH_2_O*s/L). TI, synchrony was inferior between H_N_ and H_S_ at increased settings and FM.

**Conclusions:**

H_N_ might hold some advantages with respect to interaction and synchrony between subject and ventilator, but studies on patients are needed to confirm these findings.

**Trial registration:**

ClinicalTrials.gov NCT01610960

## Introduction

The choice of the interface is one of the crucial factors determining the success of noninvasive ventilation (NIV) in both the acute [1] and chronic [2] setting. Different types of face mask (FM) that is, oral, nasal, oronasal, total full face of different size, design, and material are available to increase the patient’s comfort. Despite the broad availability of FMs, most NIV failures are still associated with mask-related side effects such as air leaks [3], skin breakdown [4,5], and mask discomfort [1,2,6].

New interfaces have been introduced with the aim of improving patient comfort and overcoming these side effects. The helmet is a transparent latex-free polyvinylchloride hood; in its standard configuration it is joined by a rigid plastic ring to a soft collar and secured by two padded armpit braces at four hooks (two in the front and two in the back of the plastic ring). The standard helmet (H_S_) has been shown to improve NIV comfort over time compared to FM and to reduce skin breakdown, gastric distension, and eye irritation [7,8]. Despite these advantages, the helmet has specific drawbacks primarily related to its highly compliant soft collar [9]. Inspiratory pressure dissipation increases the time lag between inspiratory effort and ventilator assistance, worsening patient ventilator synchrony [10-12]. Furthermore, compared to FM the helmet has been found to be associated with less efficient inspiratory-muscle unloading, both in healthy volunteers [11] and patients [10,12]. In addition, the armpit braces that maintain the helmet in place may cause patients discomfort and skin lesions leading to NIV intolerance and failure [13].

To reduce these technical problems, a new helmet (H_N_) (NIV-Castar R Next, Starmed, Mirandola, Italy) has been developed and introduced into clinical use, in which an opening ring placed underneath an inflatable cushion secures the helmet without the need for armpit braces. Moreover, compared to H_S_, with H_N_ the pressure dissipated because of the downward displacement of the soft collar during ventilator insufflation is eliminated, or at least reduced to a large extent. Very recently a bench study comparing H_S_ and H_N_ suggested a better performance of the latter in terms of triggering performance, patient-ventilator synchrony, and rate of pressurization [14].

The rotational use of different interfaces has been proposed to improve patients’ tolerance and prolong NIV application both in hypoxemic [15] and hypercapnic [16] patients. On one hand, patients needing several daily hours of NIV may benefit from the rotational use of different interfaces to reduce the risk of discomfort and the side effects of one specific interface [15-17]; on the other hand, the use of diverse interfaces with equal ventilator assistance may result in a different extent of muscle unloading and patient-ventilator synchrony [10,12]. To overcome the decreased efficacy of the H_S_ in delivering ventilator assistance, compared to the FM, Vargas *et al*. recently proposed to increase the level of both positive end-expiratory pressure (PEEP) and inspiratory pressure support (PS) [12].

The objective of this physiologic study was to assess and compare the efficacy of FM, H_S_, and H_N_ in delivering NIV in healthy volunteers. Interaction and synchrony between subject and ventilator, and the subject’s inspiratory effort and comfort were compared at baseline settings, that is, at PEEP 5 cmH_2_O and PS 5 cmH_2_O, and at increased settings, that is, PEEP 8 cmH_2_O and PS 8 cmH_2_O.

## Materials and methods

The protocol was approved by the institutional review board (Comité de Protection des Personnes Sud Méditéranée IV, Montpellier; approval number 2012-A00098-35), and written informed consent was obtained from each subject. Twelve healthy volunteers were studied in a semi-recumbent position. The subjects (three women and nine men, age 30 ± 9 years) were all nonsmokers and had a body mass index of 24 ± 4 kg/m^2^. NIV was delivered trough an FM (Performatrak, Philips Respironics), H_S_ (NIV-Castar R), and H_N_ (NIV-Castar R Next), using an ICU ventilator equipped with software for air-leaks compensation (NIV module) (Servo-I, Maquet, Solna, Sweden) set in PS ventilation mode.

### Measurements

**Figure 1:**
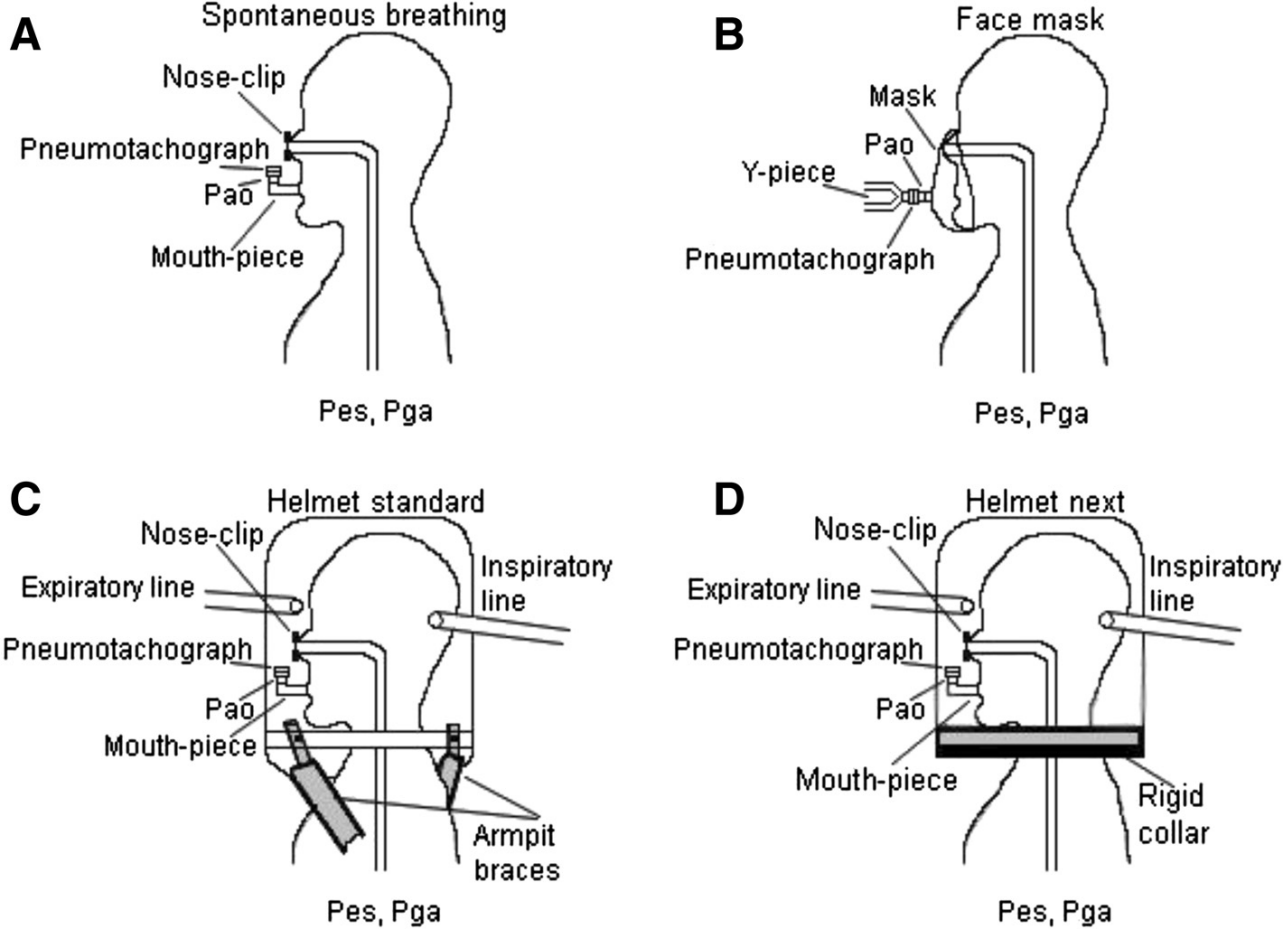
**Schematic representation of the experimental design. (A)** Spontaneous breathing; **(B)** face mask; **(C)** helmet standard; **(D)** helmet next. Pao, pressure at the airway opening; Pes, esophageal pressure; Pga, gastric pressure.

Airflow was measured with a pneumotachograph (Fleish no. 2; Fleisch, Lausanne, Switzerland) connected to a differential pressure transducer (MP45, ± 2 cmH_2_O; Validyne, Northridge, CA, USA). The pneumotachograph was placed between the mask inlet and the Y-piece of the ventilator circuit during FM, and distally to the mouthpiece during both spontaneous breathing and helmet ventilation as shown in Figure 1. Tidal volume (V_T_) was obtained by digital integration of the flow signal. Pressure at the airway opening (Pao) was assessed via a side port connected to a pressure transducer. Esophageal (Pes) and gastric (Pga) pressures were measured using a double balloon-tipped catheter (Nutrivent, Sidam s.r.l., Mirandola, Italy) positioned in the mid-esophagus and in the stomach and connected to two differential pressure transducers. The esophageal and gastric balloons were inflated with 4 ml of air. The correct positioning of the esophageal balloon was checked by an inspiratory occlusion [18]. Adequate placement of the gastric balloon was ascertained by gentle manual pressure on the subject’s abdomen to observe fluctuations in gastric pressure, as well as by asking the patient to swallow and verifying that the sharp increase in the esophageal pressure caused by esophageal contraction was not observed on the Pga tracing. Transdiaphragmatic (Pdi) pressure was obtained by subtracting Pes from Pga.

Patient’s own (neural) respiratory rate (RRn) and ventilator rate of cycling (RRv) were determined from Pdi and Pao swings, respectively. In line with previous studies [10,11,19,20], the patient’s inspiratory time (TI,p) was estimated from the Pdi tracing as the time between the onset of the positive Pdi swing above baseline (that is, the start of inspiratory effort) and the point where Pdi started to fall toward baseline. Likewise, the duration of the inspiratory assistance provided by the ventilator (TI,v) was calculated from the Pao tracing. The inspiratory trigger delay (Delay,TR-insp) was calculated as the time lag between onset of inspiratory effort and start of ventilator support, and the expiratory trigger delay (Delay,TR-exp) was calculated as the time lag between the points at which Pdi and Pao started to fall toward baseline. We also calculated the time of synchrony between muscle effort and ventilator support (TI,p-TI,v synchrony) as the period in the course of inspiration during which the diaphragm was contracting and the ventilator was concurrently delivering support.

The pressure-time product of the transdiaphragmatic (PTPdi) pressure was assessed to determine the effort exerted by the diaphragm and to provide a surrogate estimate of the overall inspiratory muscle exertion, not considering the amount of effort spent to distend the chest wall, from the changes in Pdi over time. The pressure-time product was calculated per minute, determining the area under Pdi (PTPdi/min) within a 1-minute time interval, per breath (PTPdi/br), dividing PTPdi/min by RRn, and per liter, dividing PTPdi/min by minute ventilation (V_E_) [10,12,21].

The inspiratory work of breathing (WOB) performed by the patient was computed from Pes and V_T_ loops as previously described [22,23]. Briefly, the inspiratory work per breath was calculated from the Campbell diagram by computing the area enclosed between the inspiratory esophageal pressure-V_T_ curve on the one hand, and the static esophageal pressure-volume curve of the chest wall on the other hand, using a theoretical value for chest-wall compliance (4% of the predicted value of the vital capacity per cm H_2_O). Although we directed careful attention to minimizing leaks around the mask, this problem did occur and was taken into consideration. Because leaks around the mask are more likely to occur during inspiration, leading to overestimation of the inspired volume, we calculated inspiratory WOB by applying a correction factor to the inspiratory flow based on the patient’s expired minute volume [23,24]. For this, we measured the ratio of expired over inspired minute volume and applied a correction factor equal to this ratio to the flow signal used to measure inspiratory WOB. Inspiratory WOB was expressed as the work per breath (J/breath), as the work per volume unit (J/L), and as the work per time unit (J/min).

Comfort was assessed at the end of each trial, using a 0- to 10-item visual analog scale [25,26] (0 = intolerable discomfort, 3 = strong discomfort, 8 = moderate discomfort, 10 = no discomfort). Heart rate and pulse arterial oxygen saturation were monitored throughout the protocol.

### Experimental protocol

**Figure 2:**
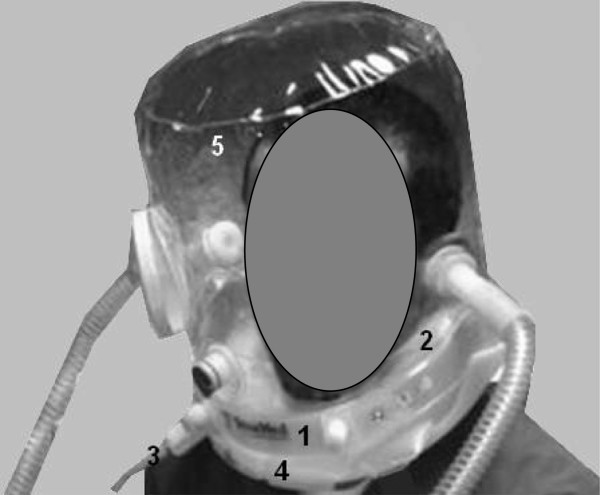
**Helmet next.** The new helmet (Helmet next) is composed of a rigid plastic ring (1), inflatable cushion (2) with its inflating line (3), anular opening ring secured to the rigid ring underneath the cushion (4), and transparent hood (5). The helmet is secured to the head by the inflated cushion placed around the neck below the jaw.

A trial of spontaneous unassisted breathing (SB) was performed in all subjects at the beginning of the study protocol (SB_I_), followed by three NIV trials with FM, H_S_, H_N_ at baseline settings (PEEP and PS set at 5 cmH_2_O) and two additional trials with the increased settings (PS and PEEP set at 8 cmH_2_O) for H_S_ and H_N_ only. In all five NIV conditions, each period lasted 5 minutes. The interfaces were randomly applied following a computer-generated random sequence. Once the interface was determined a second computer-generated random sequence was applied to establish the setting. To conclude, a second trial of spontaneous unassisted breathing was performed (SB_E_). A schematic representation of the experimental setup used during unassisted spontaneous breathing, FM, H_S_ and H_N_ ventilation is shown in Figure 1, and the new helmet is depicted in detail in Figure 2.

The ICU ventilator Servo-i was set in NIV mode with an inspired oxygen fraction (FiO_2_) of 21%, the fastest rise time, an inspiratory trigger at five units, corresponding to one l/minute, and cycling-off flow threshold of 25% of the peak inspiratory flow. These last settings were maintained unmodified throughout the study.

During the SB, the H_S_, and H_N_ trials, the subjects wore a nose clip and breathed through a mouthpiece connected to a side port connector for the measurement of the pressure at the airway opening, and to a pneumotachograph. The measuring equipment had a dead space of 49 ml and resistance of 2.0 and 2.6 cmH_2_O L^-1^ s^-1^ at a flow rate of 0.5 and 1.0 L/s, respectively.

### Data analysis

After elimination of cycles contaminated by artifacts due to cough and esophageal spasms, 20 consecutive breaths, closest to the end of the 5-minute run, were used to compute average values. All observations were included in the analysis. The signals were amplified, low-pass filtered, digitized at 128 Hz and sampled using an analog-to-digital converter system (MP100; Biopac Systems, Santa Barbara, CA, USA). Results are expressed as means ± SD and as group-mean difference ± SD. Normality of quantitative variables (Shapiro-Wilk test) was checked. Data were compared using a generalized linear mixed-effects model for repeated measures, taking into account repeated measures as random effects, and NIV condition, cross-over sequence and its interaction as fixed effects. Normality of residuals was checked after applying the linear mixed-effects model. Comparisons defined a priori were FM versus H_S,_ FM versus H_N,_ SB_I_ versus H_S,_ and SB_I_ versus H_N._ Significance was set at *P* <0.0125 after correction using the Bonferroni *t*-test for the number of multiple comparisons, that is, four.

## Results

### Vital signs and comfort

No subjects asked to discontinue the trial for any reason. Heart rate and pulse arterial oxygen saturation remained unchanged, regardless of interface and ventilator setting (data not shown). Increasing Pao produced a progressive decrease in patient comfort that wasd statistically significant only between SB, FM and increased settings. In fact, comfort was scored 8.9 ± 1.3 during SB and 7.7 ± 1.1, 7.5 ± 1.4, and 7.4 ± 1.8 at baseline settings during FM, H_S_, and H_N_, respectively, whereas the scores were 6.4 ± 1.6 and 6.7 ± 1.6 at increased settings for H_S_ and H_N_, respectively. Group-mean differences between FM and H_S_, FM and H_N,_ SB and H_S_, SB and H_N_ for comfort were 1.4 ± 1.1, 1.0 ± 1.4, 2.5 ± 1.3, and 2.2 ± 1.2, respectively. Of note, there was no significant difference between the interfaces on iso-assistance.

Table 1 presents the baseline data and Table 2 the data following intervention at increased settings, with group-mean differences between FM and SB versus H_S_ and H_N_ presented as mean ± SD (see Table E1 in Additional file 1 in which data are presented as median (interquartiles) in the appendix).

**Table 1:** Main ventilatory variables and inspiratory efforts at baseline

	**SB**_**I**_	**Baseline settings**			**SB**_**E**_
		**FM**	**H**_**S**_	**H**_**N**_	
**V**_**T **_**ml**	787 ± 220	596 ± 147	794 ± 250	821 ± 240	842 ± 275
**RRn breath/minute**	13 ± 6	15 ± 7	14 ± 5	14 ± 5	14 ± 6
**V**_**E **_**L/minute**	9.6 ± 3.9	8.1 ± 2.3	10.2 ± 2.7	11.1 ± 4.9	10.7 ± 4.1
**PTPdi/breath**	20.0 ± 10.4	17.0 ± 11.0	16.6 ± 10.0	15.7 ± 10.7	17.4 ± 15.6
**PTPdi/minute**	241 ± 117	204 ± 81	201 ± 92	198 ± 109	190 ± 116
**PTPdi/L**	27.6 ± 15.5	30.2 ± 16.8	21.6 ± 10.5	20.9 ± 13.2	20.4 ± 14.8
**WOB J/breath**	0.456 ± 0.214	0.335 ± 0.142	0.400 ± 0.302	0.447 ± 0.309	0.460 ± 0.379
**WOB J/minute**	5.61 ± 2.71	4.54 ± 2.49	5.11 ± 3.00	6.60 ± 7.49	5.53 ± 3.98
**WOB J/L**	0.585 ± 0.206	0.525 ± 0.188	0.494 ± 0.172	0.569 ± 0.277	0.504 ± 0.274
**Delay,TR-insp (s)**	NA	0.181 ± 0.086	0.354 ± 0.081	0.276 ± 0.091	NA
**Delay,TR-exp (s)**	NA	0.275 ± 0.038	0.395 ± 0.093	0.301 ± 0.044	NA
**TI,Synchrony (%)**	NA	90 ± 4	79 ± 7	83 ± 4	NA

Data are presented as mean ± SD. Baseline data; n = 12 healthy volunteers included. Baseline settings for FM, H_S_, H_N_: PEEP 5 cmH_2_O; PS 5 cmH_2_O, Ramp 0; Exp trigger: 25%; FiO_2_: 21%. V_T_, tidal volume; V_E_, minute ventilation; RRn, neural respiratory rate; PTPdi, pressure-time product of the transdiaphragmatic pressure; WOB, work of breathing; FM, face mask; H_S_, helmet standard; H_N_, helmet next; SB_I_, initial spontaneous breathing; SB_E_, end spontaneous breathing; PEEP, positive end-expiratory pressure; PS, pressure support; Exp trigger, expiratory trigger; delay, TR-insp, inspiratory trigger delay; Delay,TR-exp, expiratory trigger delay; TI,Synchrony, synchrony time as% of patient inspiratory time; NA, not applicable.

**Table 2:** Main ventilatory variables and inspiratory effort following interventions, at increased settings, with group means differences between FM and SB versus HS and HN

	**Increased settings**		**Group mean difference (95% CI)**			
	**H**_**S**_	**H**_**N**_	**FM-H**_**S**_	**FM-H**_**N**_	**SB**_**I**_**-H**_**S**_	**SB**_**I**_**-H**_**N**_
**V**_**T **_**ml**	882 ± 276	853 ± 278	-286 ± 338*	-257 ± 320*	-95 ± 219	-67 ± 222.
**RRn breath/minute**	13 ± 4	14 ± 5	1.9 ± 4.5	0.42 ± 3.4	0.42 ± 3.4	-1.08 ± 3.3
**V**_**E **_**L/minute**	10.6 ± 3.0	11.6 ± 4.7	-2.7 ± 1.9*	-3.6 ± 3.1*	-1.0 ± 2.5	-2.0 ± 3.1
**PTPdi/breath**	13.0 ± 10.5	10.7 ± 9.9	4.0 ± 5.6*	6.3 ± 7.4*	7.0 ± 9.4*	9.4 ± 10.1*
**PTPdi/minute**	142 ± 80	128 ± 96	6.8 ± 93.2	75.6 ± 82.7*	44.2 ± 128.7*	113.1 ± 119.2*
**PTPdi/L**	15.2 ± 10.0	12.6 ± 9.9	9.3 ± 14.5	17.6 ± 13.7*	6.7 ± 15.6	15.0 ± 14.7*
**WOB J/breath**	0.412 ± 0.313	0.390 ± 0.314	-0.11 ± 0.33	-0.05 ± 0.35	0.01 ± 0.34	0.07 ± 0.33
**WOB J/minute**	5.04 ± 4.10	5.75 ± 5.79	-2.1 ± 5.9	-1.1 ± 4.6	-1.1 ± 5.6	-0.04 ± 4.0
**WOB J/L**	0.448 ± 0.233	0.446 ± 0.267	0.02 ± 0.24	0.12 ± 0.25	0.06 ± 0.29	0.17 ± 0.30
**Delay,TR-insp (s)**	0.345 ± 0.073	0.230 ± 0.061	-0.16 ± 0.09	-0.05 ± 0.08	NA	NA
**Delay,TR-exp (s°**	0.367 ± 0.074	0.290 ± 0.099	-0.09 ± 0.08	-0.02 ± 0.11	NA	NA
**TI,Synchrony (%)**	80 ± 5	85 ± 2	9.8 ± 5.9*	4.2 ± 4.1*	NA	NA

Data are presented as mean ± SD and group mean differences ± SD. Data following intervention; n = 12 healthy volunteers included. Increased settings for H_S_ and H_N_ were PEEP 8 cmH_2_O; PS 8 cmH_2_O, Ramp 0; Exp trigger: 25%; FiO_2_: 21%. Data were analyzed with a generalized linear mixed-effects model for repeated measures, ^*^P <0.0125. V_T_, tidal volume; V_E_, minute ventilation; RRn, neural respiratory rate; PTPdi, pressure-time product of the transdiaphragmatic pressure; WOB, work of breathing; FM, face mask; H_S_, helmet standard; H_N_, helmet next; SB_I_, initial spontaneous breathing; SB_E_, end spontaneous breathing; PEEP, positive end-expiratory pressure; PS, pressure support; Exp trigger, expiratory trigger; delay, TR-insp, inspiratory trigger delay; Delay,TR-exp, expiratory trigger delay; TI,Synchrony, synchrony time as% of patient inspiratory time; NA, not applicable.

### Breathing pattern and inspiratory effort

**Figure 3:**
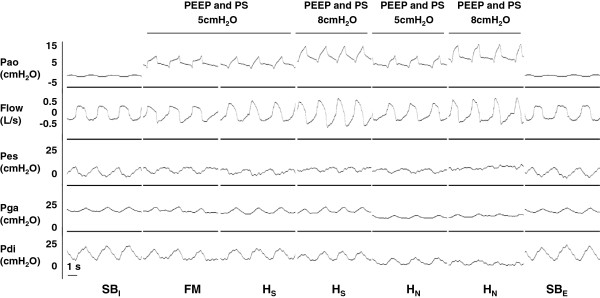
**Representative traces of a patient (patient 1) during the randomized trials for spontaneous breathing, face mask at PEEP and PS of 5 cmH**_**2**_**O, helmet standard at PEEP and PS of 5 cmH**_**2**_**O, helmet standard at PEEP and PS of 8 cmH**_**2**_**O, helmet next at PEEP and PS of 5 cmH**_**2**_**O, helmet next at PEEP and PS of 8 cmH**_**2**_**O, and spontaneous breathing.** Pao, Flow, Pes, Pga, and Pdi are shown from top to bottom. FM, face mask; H_N_, helmet next; H_S_, helmet standard; Pao, pressure at the airway opening; Pdi, transdiaphragmatic pressure; PEEP, positive end-expiratory pressure; Pe, esophageal pressure; Pga, gastric pressure; PS, pressure support.

**Figure 4:**
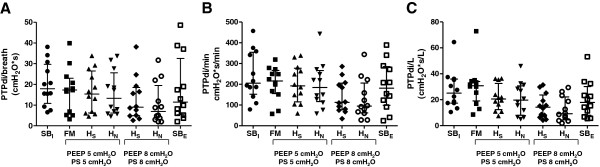
**The pressure**-**time product of the transdiaphragmatic pressure calculated per breath, per minute, and per liter. (A)** Calculation per breath; **(B)** calculation per minute; **(C)** calculation per liter. FM: face mask; HN: helmet next; HS: helmet standard; PEEP: positive end-expiratory pressure; PS: pressure support; PTPdi: pressure-time product of the transdiaphragmatic pressure; SB: spontaneous breathing.

An example of illustrative traces, that is, Pao, flow, Pes, Pga, and Pdi recorded during the experimental trials, is depicted in Figures 3 and 4.

Expiratory V_T_ was lower, with FM, as opposed to all other conditions. The differences in the respiratory rate (RR) were small and not significant. Minute ventilation (V_E_) significantly increased with H_N_ at baseline settings (*P* <0.0125) and with both helmets at increased settings (*P* <0.0125) compared to FM.

Compared with SB_I_, NIV administered at baseline settings did not modify inspiratory effort, as assessed by pressure-time product of PTPdi/breath, PTPdi/min and PTPdi/L, irrespective of the interface applied. H_N_ at increased settings performed better than FM in decreasing inspiratory effort measured by PTPdi/breath (10.7 ± 9.9 versus 17.0 ± 11.0 cmH_2_O*s), PTPdi/min (128 ± 96 versus 204 ± 81 cmH_2_O*s/min), and PTPdi/L (12.6 ± 9.9 versus 30.2 ± 16.8 cmH_2_O*s/L). There was no significant difference in WOB irrespective of support level and type of interface applied.

### Patient-ventilator synchrony

At baseline settings, Delay,TR-insp was significantly shorter (*P* <0.0125) with FM (0.181 ± 0.086 s), compared to both H_S_ and H_N_ (0.354 ± 0.081 s and 0.276 ± 0.091 s, respectively). At increased settings, Delay,TR-insp was shorter (*P* <0.0125) with H_N_ (0.230 ± 0.061 s), compared to H_S_ (0.345 ± 0.073 s). With both H_S_ and H_N_ the increased settings decreased Delay,TR-insp, which, however, was not different to FM with H_N_ only.

At baseline settings, Delay,TR-exp was similar between FM (0.275 ± 0.038 s) and H_N_ (0.301 ± 0.044 s), whereas for H_S_ (0.395 ± 0.093 s) it was significantly longer (*P* <0.0125). Although at increased settings Delay,TR-exp slightly improved with both H_N_ (0.290 ± 0.099 s) and H_S_ (0.367 ± 0.074 s), with the latter it remained significantly longer, as opposed to FM.

Time of synchrony (TI,synchrony), expressed as percent of patient’s inspiratory time, was longer during FM (90 ± 4%), compared to H_S_ (79 ± 7%) (*P* <0.0125) and H_N_ (83 ± 4%) at baseline settings. At increased settings, no difference was found between helmets, but compared to FM, TI,synchrony remained significantly lower with H_S_ (80 ± 5%) and H_N_ (85 ± 2%).

## Discussion

We found that in healthy volunteers FM, H_S_ and H_N_ at baseline settings, that is, PEEP 5 cmH_2_O and PS 5 cmH_2_O, were not significantly different with respect to inspiratory muscles unloading, WOB and comfort. With respect to patient-ventilator interaction, TI,synchrony and Delay,TR-insp were significantly improved by FM compared to both helmets, whereas Delay,TR-exp was shorter with FM, as opposed to H_S_, but not H_N_. At increased settings (that is, PEEP 8 cmH_2_O and PS 8 cmH_2_O), inspiratory effort was significantly lower, compared to SB_I_ and FM, with H_N_, while not HS. Comfort was inversely related to the pressure applied to the airway opening by the ventilator, regardless of the interface. Patient-ventilator synchrony, as assessed by Delay,TR-insp and Delay,TR-exp, was equivalent between FM at baseline setting and H_N_ at increased settings, but remained inferior with H_S_ even at increased settings. TI synchrony remained inferior with both helmets at increased settings, compared to FM.

Before discussing our findings, some limitations of this study merit consideration. First, our investigation was conducted on healthy individuals, which makes our findings not entirely inferable to the clinical setting. We share this limit with several studies, some of which were performed to evaluate H_S_ at the time it was introduced into clinical use [9,11,21]. As a matter of fact, studies on H_N_ are lacking, which justifies this first *in vivo* evaluation in healthy volunteers. Second, of the seven trials performed on each subject, the two spontaneous breathing trials and four helmet trials had the same breathing apparatus with mouth-piece and nose-clip, whereas the FM trial did not. The use of breathing apparatus that is, mouth-piece and nose-clip, has been repeatedly shown to affect breathing pattern by increasing V_T_ and V_E_ with few changes in the RR [27,28]. In keeping with these studies, during the FM trial V_T_ and V_E_ were smaller than during the other six trials, the reduction in V_E_ being statistically significant, whereas the RR was only slightly decreased. As V_T_ is one of the variables, in addition to Pes, computed in the measurement of WOB, the use of a breathing apparatus might have influenced it to a similar extent. Third, we limited the time of each trial to 5 minutes, which is indeed rather short, especially when compared to that utilized in other similar studies performed either in healthy volunteers [9,21] or patients [10,12]. It should be considered, however, that in contrast to these studies, in our experimental set up the breathing apparatus was connected to the subject through the mouthpiece, which produces discomfort, potentially affecting either the breathing pattern or the indexes of respiratory muscle effort. Although we cannot exclude that the relatively short period of evaluation could be not sufficient to fully achieve a steady state, we are convinced that the poor tolerance to the breathing apparatus would have represented more bias. Finally, potential bias related to the non-blinded setting of the study has to be taken into consideration, although we share this limitation with several studies [9,11,21].

In hypercapnic patients with severe chronic obstructive pulmonary disease, Navalesi *et al*. observed a significant decrease per minute in PTPdi of 65% and 43% with FM and H_S_, respectively, as opposed to SB [10,12]. In patients at high risk of developing post-extubation respiratory failure, Vargas *et al*. found that at baseline settings, FM and H_S_ significantly reduced PTPdi per minute compared to SB, at 69% and 60%, respectively [12]. In our healthy volunteers, we also observed a reduction in PTPdi per minute at baseline settings with respect to SB, but the extent of this decrease range was lower (15 to 20%) and not statistically significant, or significantly different between the three interfaces. On one hand, these discrepancies are likely a consequence of the lower inspiratory pressure applied and on the other, due to the fact that in contrast to the aforementioned studies [10,12], we performed our investigation in normal subjects without underlying respiratory disorders. Indeed, other previous studies on healthy subjects at similar baseline settings also failed to demonstrate a difference in PTPdi between FM and H_S_[11], and between H_S_ and SB [21]. In keeping with Vargas *et al*., we found that at increased settings H_S_ and H_N_ were both as effective as FM at the baseline setting, in reducing inspiratory effort compared to SB; of note, PTPdi expressed per breath, per minute or per liter, was significantly lower with H_N_ at increased settings than with FM at baseline settings.

Surprisingly, the differences in WOB between trials were rather small and not significant. The large V_T_, that to some extent was a consequence of the breathing apparatus [27,28] as already mentioned, may have influenced this variable, which is influenced by both Pes and V_T_. This may also explain the lower, though not significantly lower values of WOB/breath and WOB/minute observed during the FM trial, in contrast to WOB/L, which, in fact was not influenced by the amount of V_T_. A possible expiratory activation of the expiratory respiratory muscles may contribute to the better performance of both H_S_ and H_N_ compared to the FM.

In agreement with previous work [10,12], at baseline settings both Delay,TR-insp and Delay,TR-exp were longer with H_S_ than with FM, whereas with H_N_ Delay,TR-insp, but not Delay,TR-exp, was significantly higher, with respect to FM. Whereas Vargas *et al*. found that Delay,TR-insp with H_S_ was significantly lower with the increased settings than with the baseline settings, in our study H_S_ at increased settings compared to H_S_ at baseline settings did not produce significant improvements in Delay,TR-insp, Delay,TR-exp or TI,synchrony, which all remained worse than the corresponding values obtained with FM at standard settings. Conversely, with H_N_ the increased settings improved all three variables, which were not significantly different compared to FM at baseline.

H_S_ was shown to be better tolerated than FM in studies comparing the two interfaces over the medium (hours) and long (days) period [7,8]. In the present investigation, comfort was not influenced by the type of interface, as already reported in the previous short term physiologic investigations comparing FM and H_S_ over brief periods of time (20 to 30 minutes) [10,12], which has been attributed to the longer time required for most of the major determinants of patient discomfort during NIV to take place [10,12]. Consistent with previous work on healthy volunteers [21], however, we found that comfort was affected by the amount of applied pressure.

Very recently, studies conducted either in hypoxemic [15] and hypercapnic [16] patients, and a European survey [17] on the use of NIV, indicate the rotational use of different interfaces as a possible means to improve patients’ tolerance and prolong NIV application. Our study shows that different interfaces can be used to achieve comparable results with respect to inspiratory effort reduction. In addition, we found that in healthy volunteers, although FM performs better overall than helmets at baseline settings, at increased settings both helmets had improved performance and resulted in lower inspiratory effort lower than SB with both helmets, and even better than FM with H_N_ only. Furthermore, at increased settings H_N_ achieved better results for interaction and synchrony between subject and ventilator compared to H_S_, which indicated that this new interface performed similarly to FM.

## Conclusions

H_N_ might hold some advantages compared to the other interfaces with respect to interaction and synchrony but studies on patients are needed to confirm these findings in the clinical setting.

## Key messages

•H_N_ is a new interface that ameliorates its previous standard version, H_S_, by reducing the pressure dissipated because of the downward displacement of the soft collar during ventilator insufflation, and by avoiding the use of armpit braces.

•We found that NIV via an FM, H_S_ and H_N_ at baseline settings, that is, PEEP 5 cmH_2_O and PS 5 cmH_2_O, were not significantly different with respect to inspiratory muscles unloading, WOB and comfort in healthy volunteers. With respect to patient-ventilator interaction and inspiratory trigger delay synchrony time and were significantly improved by FM, compared to both helmets, whereas expiratory trigger delay was also shorter with FM, as opposed to H_S_, but not H_N_.

•At increased settings with H_N_ (that is, PEEP 8 cmH_2_O and PS 8 cmH_2_O), inspiratory effort was significantly lower compared to that obtained while breathing spontaneously without assistance and FM_._ Patient-ventilator synchrony, as assessed by inspiratory trigger delay and expiratory trigger delay was equivalent between FM at baseline setting and H_N_ at increased settings, but remained inferior with H_S_ even at increased settings.

## Abbreviations

Delay,TR-exp: expiratory trigger delay; Delay,TR-insp: inspiratory trigger delay; FiO2: inspired oxygen fraction; FM: face mask; HN: helmet next; HR: heart rate; HS: helmet standard; NIV: nonivasive ventilation; Pao: pressure at the airway opening; Pdi: transdiaphragmatic pressure; PEEP: positive end-expiratory pressure; Pes: esophageal pressure; Pga: gastric pressure; PS: pressure support; PSV: pressure support ventilation; PTPdi: pressure-time product of transdiaphragmatic pressure; RR: respiratory rate; RRn: patient’s neural respiratory rate; RRv: ventilator rate of cycling; SB: spontaneous breathing; SBE: end spontaneous breathing; SBI: initial spontaneous breathing; SpO2: pulse arterial oxygen saturation; TI,p: patient’s inspiratory time; TI,synchrony: time of synchrony; TI,v: ventilator’s inspiratory time; VAS: visual analog scale; VE: minute ventilation; VT: tidal volume; WOB: work of breathing.

## Competing interests

StarMed financially contributed by providing helmets (NIV-Castar R Next and NIV-Castar R) and double balloon-tipped catheters. PN contributed to the development of the helmet NIV-Castar R Next, for which the licence for the patent belongs to Starmed SPA, and receives royalties for that invention. SJ was a paid consultant to Starmed SPA. RV, ADJ, MC, FG, MM, YC, AP have no conflicts of interest to declare.

## Authors’ contributions

RV contributed to the conception and design of the study, to the analysis and interpretation of data, to drafting the submitted article, and to providing final approval of the version to be published. ADJ contributed to the analysis of the data, and to providing final approval of the version to be published. MC contributed to the acquisition of the data, and to providing final approval of the version to be published. FG contributed to the acquisition of the data, and to providing final approval of the version to be published. MM contributed to the acquisition of the data, and to providing final approval of the version to be published. YC contributed to the acquisition of the data, and to providing final approval of the version to be published. PA contributed to the acquisition of the data, and to providing final approval of the version to be published. PN contributed to the conception of the study, interpretation of data and drafting of the submitted article, and to providing final approval of the version to be published. SJ contributed to the conception and design of the study; to the analysis and interpretation of data; to drafting the submitted article, and to providing final approval of the version to be published. All authors read and approved the final manuscript.

## Supplementary Material

Additional file 1: Tables E1 and E2 An additional file in the appendix contains two additional tables: **Table E1** shows the main ventilatory variables and inspiratory effort parameters obtained at baseline settings and following interventions at increased settings, obtained in spontaneous breathing (SB), facial mask (FM), standard helmet (HS) and helmet next (HN) with data presented as median [interquartiles]; and **Table E2** shows the inspiratory and expiratory trigger delay and synchrony time obtained at baseline settings and following interventions at increased settings, obtained in facial mask (FM), standard helmet (HS) and helmet next (HN) with data presented as median [interquartiles]. Click here for file
