# Supplementary material for: Comparative evaluation of three interfaces for non-invasive ventilation: a randomized cross-over design physiologic study on healthy volunteers
Source: Crit Care. 2014 Jan 3;18(1):R2. doi: 10.1186/cc13175 (PMC4056758; doi:10.1186/cc13175)
Supplement: Additional file 1: Tables E1 and E2 — An additional file in the appendix contains two additional tables: Table E1 shows the main ventilatory variables and inspiratory effort parameters obtained at baseline settings and following interventions at increased settings, obtained in spontaneous breathing (SB), facial mask (FM), standard helmet (HS) and helmet next (HN) with data presented as median [interquartiles]; and Table E2 shows the inspiratory and expiratory trigger delay and synchrony time obtained at baseline settings and following interventions at increased settings, obtained in facial mask (FM), standard helmet (HS) and helmet next (HN) with data presented as median [interquartiles]. [file cc13175-S1.docx]

**Additional file 1**

**Title: Comparative evaluation of three interfaces for non-invasive ventilation: a randomized cross-over design physiologic study on healthy volunteers.**

Version 24 Nov 2013

Rosanna Vaschetto^1,2^ MD, PhD, Audrey De Jong^2^ MD, Matthieu Conseil MD^2^,

Fabrice Galia^2^, Martin Mahul^2^ MD, ^2^ MD, Yannael Coisel^2^ MD, Albert Prades^2^,

Paolo Navalesi^3,4,5^ MD, Samir Jaber^2^ MD, PhD.

^1^Anesthesia and Intensive Care Medicine, Maggiore della Carità Hospital, Novara, Italy.

^2^Intensive Care Unit and Transplantation, Critical Care and Anesthesia Department (DAR B), Hôpital Saint-Éloi, CHU de Montpellier, Montpellier, France.

^3^Dipartimento di Medicina Traslazionale, Università del Piemonte Orientale “Amedeo Avogadro”, Alessandria-Novara-Vercelli, Italy.

^4^Anesthesia and Intensive Care Medicine, Sant’Andrea Hospital, Vercelli, Italy.

^5^CRRF Mons. L. Novarese, Moncrivello, Vercelli, Italy.

An additional file in appendix contains two additional tables: Table E1 shows the main ventilatory variables and inspiratory efforts parameters obtained at baseline settings and following interventions at increased settings, obtained in spontaneous breathing (SB), facial mask (FM), standard helmet (HS) and helmet next (HN) with data presented as median [interquartiles]; and Table E2 shows the inspiratory and expiratory trigger delay and synchrony time obtained at baseline settings and following interventions at increased settings, obtaned in facial mask (FM), standard helmet (HS) and helmet next (HN) with data presented as median [interquartiles].

|  | **SB_I_** | **Baseline Settings** | | | **Increased Settings** | | **SB_E_** |
| --- | --- | --- | --- | --- | --- | --- | --- |
|  |  | **FM** | **H_S_** | **H_N_** | **H_S_** | **H_N_** |  |
| **V_T_ ml** | 730[591-967] | 579[503-662] | 760[617-885] | 773[648-1012] | 827[625-1060] | 830[634-1073] | 747[648-1143] |
| **RRn breath/min** | 12[8-16] | 13[10-17] | 13[10-15] | 13[12-16] | 13[10-15] | 14[11-16] | 12[10-15] |
| **V_E_ L/min** | 8.1[6.2-11.1] | 8.1[6.5-9.2] | 9.4[7.7-13.3] | 9.1[8.3-14.1]† | 9.1[8.3-13.3]† | 10.0[8.1-15.0]† | 8.9[7.4-13.5] |
| **PTPdi/breath** | 17.9[10.8-29.7] | 17.3[5.6-22.9] | 15.3[6.2-26.5] | 13.2[6.1-25.6] | 9.0[4.8-18.5]* | 6.9[3.1-19.5]*† | 12.0[6.3-32.5] |
| **PTPdi/min** | 205[152-354] | 215[155-266] | 192[115-277] | 184[116-266] | 112[76-204]* | 94[61-205]*† | 181[98-284] |
| **PTPdi/L** | 22.4[17.3-36.1] | 31.3[16.9-34.2] | 19.4[11.0-35.4] | 18.4[6.7-31.4] | 13.3[6.4-25.1]*† | 8.6[3.8-23.2]*† | 15.2[6.7-32.3] |
| **WOB J/breath** | 0.493[0.243-0.609] | 0.331[0.236-0.377] | 0.337[0.240-0.430] | 0.359[0.211-0.650] | 0.282[0.165-0.599] | 0.301[0.130-0.609] | 0.393[0.173-0.535] |
| **WOB J/min** | 4.7[3.7-7.1] | 4.7[2.5-5.5] | 4.1[2.9-6.1] | 5.0[2.7-6.4] | 3.6[2.2-6.5] | 3.2[2.1-7.7] | 5.5[2.2-6.2] |
| **WOB J/L** | 0.539[0.392-0.760] | 0.509[0.355-0.676] | 0.441[0.391-0.590] | 0.538[0.336-0.675] | 0.407[0.263-0.672] | 0.363[0.226-0.585] | 0.479[0.348-0.636] |

**Table E1. Main ventilatory variables and inspiratory efforts parameters obtained at baseline settings and following interventions at increased settings, obtained in spontaneous breathing (SB), facial mask (FM), standard helmet (HS) and helmet next (HN) with data presented as median [interquartiles].**

**Table E1.** N=12 healthy volunteers included. Baseline settings for FM, H_S_, H_N_: PEEP 5cmH_2_O; PS 5cmH_2_O, Ramp 0; Exp trigger: 25%; FiO_2_: 21%. Increased settings for H_S_ and H_N_: PEEP 8cmH_2_O; PS 8cmH_2_O, Ramp 0; Exp trigger: 25%; FiO_2_: 21%. Data are presented as median [interquartiles]. Data were analyzed with Friedman test and Dunn's Multiple Comparison Test, * p<0.05 vs. SB_I_. † p<0.05 vs. FM. V_T_: tidal volume; V_E_: minute ventilation; RRn: neural respiratory rate; PTPdi: pressure-time product of the transdiaphragmatic pressure; WOB: work of breathing; FM: face mask; H_S_: helmet standard; H_N_: helmet next; SB_I_: initial spontaneous breathing; SB_E_: end spontaneous breathing; PEEP: positive end-expiratory pressure; PS: pressure support; Exp trigger: expiratory trigger.

**Table E2. Inspiratory and expiratory trigger delay and synchrony time obtained at baseline settings and following interventions at increased settings, obtained in facial mask (FM), standard helmet (HS) and helmet next (HN) with data presented as median [interquartiles].**

|  | **Baseline Settings** | | | **Increased Settings** | |
| --- | --- | --- | --- | --- | --- |
|  | **FM** | **H_S_** | **H_N_** | **H_S_** | **H_N_** |
| **Delay,TR-insp (s)** | 0.144[0.122-0.217] | 0.372[0.283-0.390]*^†^ | 0.253[0.240-0.297]* | 0.325[0.300-0.415]*^†^ | 0.228[0.171-0.254] |
| **Delay,TR-exp (s)** | 0.260[0.257-0.290] | 0.403[0.349-0.445]*^†^ | 0.301[0.274-0.345] | 0.379[0.306-0.420]* | 0.305[0.231-0.344] |
| **TI,Synchrony (%)** | 92[86-93] | 79[74-83]* | 83[81-87] | 79[75-84]* | 85[83-88] |

**Table E2.** N=12 healthy volunteers included. Baseline settings for FM, H_S_, H_N_: PEEP 5cmH_2_O; PS 5cmH_2_O, Ramp 0; Exp trigger: 25%; FiO_2_: 21%. Increased settings for H_S_ and H_N_: PEEP 8cmH_2_O; PS 8cmH_2_O, Ramp 0; Exp trigger: 25%; FiO_2_: 21%. Data are presented as median [interquartiles].

Data were analyzed with Friedman test and Dunn's Multiple Comparison Test, * p<0.05 vs. FM; ^†^ p<0.05 vs. H_N_ Increased Settings. FM: face mask; H_S_: helmet standard; H_N_: helmet next; Delay,TR-insp: inspiratory trigger delay; Delay,TR-exp: expiratory trigger delay; TI,Synchrony: synchrony time as % of patient inspiratory time.
